# Acute Effects of Whole-Body Vibration on Peripheral Blood Flow, Vibrotactile Perception and Balance in Older Adults

**DOI:** 10.3390/ijerph17031069

**Published:** 2020-02-07

**Authors:** M. H. Mahbub, Ryosuke Hase, Natsu Yamaguchi, Keiichi Hiroshige, Noriaki Harada, A. N. M. Nurul Haque Bhuiyan, Tsuyoshi Tanabe

**Affiliations:** 1 Deaprtment of Public Health and Preventive Medicine, Yamaguchi University Graduate School of Medicine, Ube, Yamaguchi 755-8505, Japan; ryosuke-hase@umin.ac.jp (R.H.); natsu@yamaguchi-u.ac.jp (N.Y.); tanabe@yamaguchi-u.ac.jp (T.T.); 2 Department of Physical Therapy, Kyushu Nutrition Welfare University, Kitakyushu, Fukuoka 800-0298, Japan; hiroshige@knwu.ac.jp; 3 Department of Nursing, Junshin Gakuen University, Graduate School of Health Sciences, Fukuoka 815-8510, Japan; harada@yamaguchi-u.ac.jp; 4 Department of Neonatology, Shaheed Sayed Nazrul Islam Medical College, Kishoreganj 2300, Bangladesh; nislammc@ac.dghs.gov.bd

**Keywords:** whole-body vibration, skin blood flow, vibrotactile perception, balance, older adults

## Abstract

Background: Non-invasive application of whole-body vibration (WBV) has the potential for inducing improvements in impaired peripheral circulation, cutaneous sensation and balance among older adults. However, relevant studies have frequently applied high magnitudes of vibration and show conflicting and inconclusive results. Therefore, we attempted to ascertain the acute responses in those parameters from exposure of thirty older subjects to WBV of three different magnitudes, defined according to ISO 2631-1 (1997). Methods: Each subject randomly underwent four sessions of intervention (three bouts of 1 min exposure with 1 min between-bout rests): WBV at 15, 20, or 25 Hz with a peak-to-peak displacement of 4 mm, or control condition. Results: Both during and after intervention, dorsal foot skin blood flow increased significantly under 20 and 25 Hz exposure conditions with greater responses under the latter condition, the magnitude of which slightly exceeded the recommended value. Plantar vibrotactile perception showed significant increases after WBV exposure with overall greater responses under higher frequencies of vibration. In contrast, no WBV-induced change in balance was observed. Conclusions: WBV at 20 Hz with a magnitude within the recommended limit can be effective in inducing enhancements in peripheral blood flow; however, the same magnitude of vibration seems insufficient in improving balance among older adults.

## 1. Introduction

Among older adults, impairments in peripheral circulation are very common and strongly associated with aging [1]. Such peripheral circulatory impairments can cause delayed wound healing or impaired healing of soft tissue injuries, chronic wounds or ulcers, and pose increased risk for morbidity, loss of independence and reduced quality of life [2,3]. On the other hand, aging-associated impairments in body balance are thought to be major contributors to falls among older adults [4]. Falls among older people are a major public health concern as being the fifth leading cause of death and leading cause of fractures, serious soft tissue injuries, head trauma or other trauma-related morbidity and hospital admissions among them [5,6,7]. It is obvious that without any preventive measures against falls among older adults, related health care costs will continue to increase with the continued aging of the world’s population.

To prevent falls among older people, a number of strategies like regular exercise, vitamin D and calcium supplementation, use of hip protectors, etc., have been suggested [8]. However, the incidence of falls and fall-related injuries continues to rise [9]. In preventing falls among aging adults, training or treatment interventions using whole-body vibration (WBV) devices have been proposed as a simple, safe, easy-to-use and non-invasive intervention modality, especially for those who may be passive or physically weak, with limited mobility or physical disability, and unable to perform conventional active, high-impact or strenuous physical exercise, or with contraindications to aerobic exercise [10]. Aging is also associated with compromised cardio-postural control and orthostatic intolerance [11,12]. Intervention with WBV has shown the potential to positively influence human peripheral circulation and improve symphathovagal balance [13,14]. However, for intervention with WBV, useful or optimum vibration parameters like frequency, amplitude and acceleration have not yet been established [13]. Moreover, despite the existence of a large number of studies investigating the effects of WBV intervention on physiological and physical changes among older adults, the relevant findings seem inconsistent and inconclusive. For example, among older individuals, a number of studies observed WBV-induced improvements in lower limb blood flow [15,16] and body balance [17,18]. On the other hand, other studies could not reveal such beneficial effects from exposure to WBV on peripheral circulation [19] or body balance [20,21,22]. Also, widely variable study protocols among the investigators complicate the interpretation of the available study results. The interpretation of the observed effects of WBV has been further complicated by the fact that a number of such studies used an intervention protocol incorporating passive vibration instead of active vibration or combined WBV exposure and control conditions with simultaneous exercise training [18,19]. All these make the differentiation of the sole contribution of active WBV on the responses in peripheral circulation and balance uncertain. On the other hand, a number of those studies exposed human subjects to relatively high magnitudes of WBV, the levels of which frequently exceeded the recommended values mentioned in the relevant standards ISO 2631-1 and EU Directive 2002/44/EU [23,24]. An intervention with the application of such high magnitudes of WBV that involves the use of human subjects is considered controversial [13].

Human body balance has a close relationship with the sensitivity of plantar cutaneous mechanoreceptors, aging-related deterioration in which is thought to be an important contributor to the impairments in gait, balance and mobility among older adults [25,26]. As reported, exposure to WBV can cause stimulations of the plantar cutaneous mechanoreceptors with changes in the sensitivity of the latter [27]. However, the available reports investigating the effects of WBV on cutaneous mechanoreceptor perception of plantar feet produced conflicting results, showing both improvements [28] and suppression [27,29] in it. Due to such inconsistent or conflicting findings, the role of WBV-induced changes in plantar cutaneous perception on balance and mobility remains inconclusive. Improved knowledge on the influence of WBV intervention on the changes in plantar cutaneous perception, with consideration of the values specified in the relevant standards should help to better understand the possibility of positive changes in body balance and movement control. Also, it would assist in the interpretation of possible mechanisms underlying such changes induced by exposure to WBV. However, to our knowledge, no study has been conducted with the measurements of responses in peripheral circulation, plantar cutaneous mechanoreceptor perception and body balance and mobility from controlled exposure to WBV in the same older subjects, with consideration of the exposure values specified in the relevant international standards.

Considering the above-mentioned issues and controversies, the aim of the current study was to examine the acute effects of intervention with short-time active WBV on foot skin blood flow (SBF) and vibrotactile perception threshold (VPT), and also balance and functional mobility among older adults. For this purpose, the subjects were exposed to WBV of three different frequencies and the vibration magnitudes were defined according to the recommendations of the existing relevant standards, ISO 2631-1 and EU Directive 2002/44/EU [23,24].

## 2. Materials and Methods 

### 2.1. Selection of Subjects

**Figure 1:**
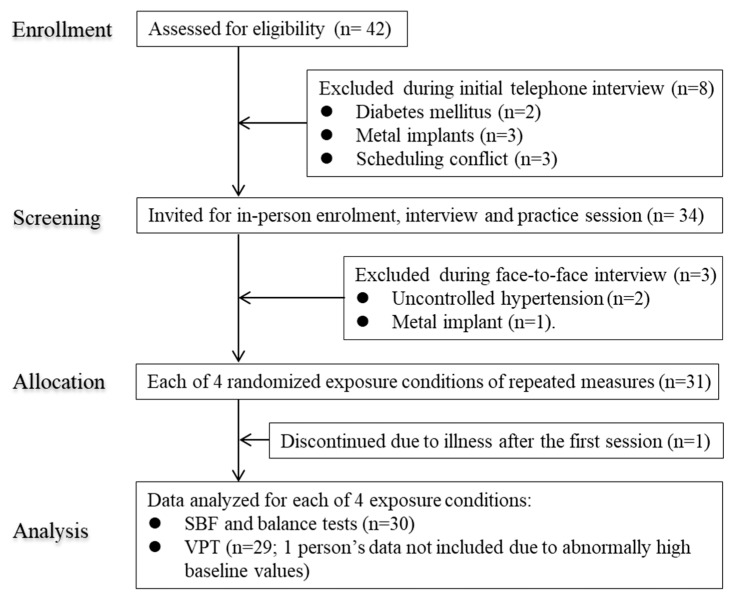
Flow diagram of participants included in this study.

This was a single-group, single blinded (participants) repeated measures study conducted among older adult subjects aged 65 years and over. The participants were recruited from nearby communities via poster advertisements and word of mouth. The selection criteria for the study subjects were as follows: non-institutionalized and able to stand; not involved in any regular exercise training with or without exposure to WBV; no reported neurological, musculoskeletal, cardiovascular (except hypertension) or connective tissue disorders or any known terminal diseases that would prohibit exposure to WBV; free from diabetes mellitus and contraindications to WBV exposure such as epilepsy, hernia, gallbladder or kidney stones; without any joint disorders or prostheses or metal implants; no history of operation within the last year, etc. A total of 42 older adult volunteers were initially invited to participate in the study (Figure 1). After exclusions, a total of 30 subjects (males 15, females 15) were finally able to complete all the experimental sessions (Figure 1). Geometric mean (95% confidence interval or CI) for age and BMI of the latter subjects were as follows: males, 73.1 (70.1 to 76.2) years and 25.7 (24.7 to 26.8) kg/m^2^, respectively; females, 71.2 (69.2 to 73.3) years and 22.5 (20.6 to 24.6) kg/m^2^, respectively.

The subjects were instructed to refrain from eating and drinking tea or coffee for at least 3 h and smoking, alcohol drinking and strenuous physical activity for at least 12 h prior to the beginning of an experimental session. They entered the experiment room after voiding. The subjects were instructed to wear light indoor clothing (two each for the upper and lower parts of the body) in the laboratory, and regular socks and shoes. Participants undergoing antihypertensive treatments were asked to adhere to their current treatment regimens during the study period. The subjects wore their regular footwear with socks during the balance tests; they were barefoot during the rest period of an experimental session.

### 2.2. Experimental Design

During all experimental sessions, the measurements of SBF were conducted before, during and after the intervention, whereas the measurements of VPT and balance and mobility tests were performed before and after the intervention.

Upon arrival, the subjects entered the first experiment room the temperature of which was maintained at around 24 °C. At first, they underwent acclimatization for a period of 15 min while seated comfortably on a height-adjustable chair. Then the balance and mobility tests were conducted as described latter.

At the end of the balance tests, the subjects moved to the next temperature-controlled (air temperature, 25.0 ± 0.5 °C) experiment room, where they were seated comfortably on another height-adjustable chair with the trouser rolled up to a level between the knees and heels, and the socks were taken off. The sensors of the thermistor were attached to the dorsal surface of the middle of left foot with adhesive tape but without tape tension not to compress the tissue. Then while seated, they underwent acclimatization for a period of 15 min without physiological or psychological stress, with the feet positioned on a wooden floor and both hands, on respective thighs. No subject had a baseline left dorsal foot skin temperature <27 °C. At the end of acclimatization period and after 5-min measurements of heart rate variability, baseline measurements of VPT were recorded. Following this, the participants were asked to stand in complete upright posture, looking forward, on the ethylene vinyl acetate foam (3 mm in thickness), fixed to the rectangular platform of the side-alternating vibration device. All the participants were allowed to grasp the rails of the platform with their hands. After a rest period of at least 3 min and ensuring a stable leg SBF, the baseline blood flow values were recorded from the dorsal region of right foot, twice, at an interval of 1 min. Then the participants were instructed to stand with knees bent to an angle of about 30° (considering a full knee extension of 0° measured by a goniometer), and position their feet 10.5 cm apart but parallel to each other on the platform. This distance was measured from the center of the platform to the midline of the heels. Then all the subjects underwent an intervention consisting of any of the following 4 exposure conditions: 1) WBV at 15 Hz, 2) WBV at 20 Hz, 3) WBV at 25 Hz, and 4) control condition (0 Hz). The peak-to-peak displacement of the vibrating platform was 4 mm. The unweighted and frequency-weighted peak accelerations were 17.75 m/s^2^ and 9.64 m/s^2^ rms, 31.56 m/s^2^; and 14.19 m/s^2^ rms, and 49.30 m/s^2^ and 17.89 m/s^2^ rms at 15 Hz, 20 Hz, and 25 Hz, respectively. The corresponding eight-hour energy-equivalent frequency-weighted acceleration or A(8) values were 0.76 m/s^2^ rms, 1.12 m/s^2^ rms, and 1.41 m/s^2^ rms, respectively, which were calculated according to the specifications mentioned in ISO 2631-1 [23]. To account for the effects of circadian rhythms and seasonal variations on physiological responses [30], we carried out the four experimental sessions randomly on four different days separated by at least 24 h, approximately at the same time for each subject, between 9.00 am and 4:00 pm during the winter season (November 2018–January 2019).

The 5-min intervention protocol consisted of three bouts of 1-min exposure separated by an interval of 1 min between the bouts. Just after the cessation of each bout of exposure, the subjects were asked to stand up quickly but smoothly and the measurements of SBF were carried out. The subjects maintained the upright posture during the 2 interval periods of 1 min each between the bouts. 

After the completion of intervention, the participants maintained the upright position for further 1 min, which was followed by the measurement of after-exposure SBF. Following this, the subjects were seated again on the chair and measurements of VPT were conducted. Then the subjects moved to the previous experiment room to undergo the balance tests after which the experimental session ended.

### 2.3. Balance and Mobility Tests

The following balance and mobility tests were performed: (a) Static balance—one leg stance (OLS) test with eyes open and closed. (b) Dynamic balance—parallel walk (PW) test and timed up and go (TUG) test. (c) Mobility and functional lower limb muscle strength—30 s chair stand (CS-30) test. All tests were performed under the supervision of an experienced experimenter who maintained a close position to the subject. The measurements and counts were performed by a trained assistant. A digital stopwatch (model 5391, Tanita, Japan) was used to record the time relevant to specific tests. For the applicable tests, the same chair without armrests (seat height, 40 cm; backrest height, 80 cm) was used by all subjects. Subjects performed two trials of each test and all the tests were presented in a random order. One min (2 min after the CS-30 test) of rest was allowed between tests with a rest period of approximately 30 s (2 min for the CS-30 test) between trials within a session when the participants were allowed to sit on a chair. A higher score denoted a better performance for assessment with OLS and CS-30 tests; for PWT and TUG tests—a higher score indicated a worse performance.

#### 2.3.1. OLS Test

The subjects maintained a standing posture with the hands holding the waist. Upon instruction (by the words “Ready, Start”), the subjects stood on one leg of preference (same for each subject for all sessions), and raised the other foot off the floor and positioned it at a level near and above the opposite ankle [31]. While standing, the subjects kept the eyes open (while looking forward on a spot marked on the front wall) or closed. The test ended if one of the following occurred: (1) the eyes were opened during eye close test; (2) the raised foot touched the ground; (3) the raised foot moved too close to or touched the opposite leg or moved away from the initial position; (4) the hands were released from the waist; (5) a subject touched the examiner for support due to the fear of falling; (6) a maximum of 45 sec elapsed [31]. The time interval from the beginning till the end of the test was recorded.

#### 2.3.2. PW Test

The subjects stood just behind the line marking the beginning of parallel lines. Upon instruction (by the words “Ready, Go”), the subjects were required to walk forwards between 2 parallel lines 6 m long and 20 cm apart [32], parallel with a wall, marked with white tape (2 cm wide) placed on the floor, with the nearest line approximately 25 cm away from the wall. The subjects were asked to use their usual gait and speed. The time for each subject to complete the 6-m walk was recorded.

#### 2.3.3. TUG Test

The subjects were seated on the standard armless chair with their arms resting on respective thighs, feet shoulder-width apart, knee flexed at 90 degrees. While instructed by the words “Ready, Go”, the subjects needed to rise from the chair, walk a distance of 3 m at a fast but safe speed without any physical assistance, cross a line and turn around a cone clockwise, walk back to the chair, and sit down again [33]. Recording of time started when a participant’s buttocks left the seat and ended when the buttocks touched the seat after completion of the walk.

#### 2.3.4. CS-30 Test

The subjects were seated with back in an upright position on the same chair mentioned above folding their hands across the chest, knee flexed at 90 degrees, feet shoulders width apart and kept flat on the floor [34]. While instructed by the word “Ready, Go”, they were required to rise from the chair till fully extended standing position and sit down again as quickly as possible. The number of complete repetitions comprising standing up and sitting down was recorded.

### 2.4. Equipment and Measurements

WBV was produced using a commercially available side-alternating vibration device (Galileo 900, Novotec Medical GmbH, Pforzheim, Germany). This WBV generating device is widely used for research (investigation of its effects on proprioception, joint position sense, leg balance, gait and functional mobility, changes in bone mineral density, muscle strength and muscle oxygenation, peripheral circulation, etc.) and rehabilitation purposes [13,35]. The peak vibration acceleration level of the unoccupied platform in the vertical (Z axis) direction was confirmed with a triaxial piezoelectric accelerometer (PV-97C, Rion Co. Ltd., Tokyo, Japan) connected to a 3-axis vibration meter (VM-54, Rion Co. Ltd., Tokyo, Japan) and fixed on it at the midpoints of the location of placement of subject’s feet.

SBF of dorsal right foot was recorded by a non-contact method using a commercially available Laser Speckle Flowgraphy system (LSFG-ANW, Softcare Co., LTD., Fukuoka, Japan), following the manufacturer’s instructions. The usefulness of this measurement modality in the evaluation of foot SBF has been described in the literature [36]. The recording unit of LSFG was firmly attached to the movable arm of a stand fixed on the floor and positioned 19 cm above the right foot of the subject at an angle of 20°. LSFG can measure the real-time SBF noninvasively as the relative velocity of the red blood cells moving through the vessels of the skin and produce a two-dimensional map of perfusion. This measurement modality incorporates a charge coupled device (CCD) camera (resolution, 600 × 480 pixels) that can capture the fluctuation in the random interference pattern (speckle pattern) of laser light reflected from the tissues. The values of blood flow indicated by the LSFG system is a relative value expressed in arbitrary units (Mean Blur Rate or MBR) as the time-averaged moving speckle pattern using the recorded frame-shaped flows for 4 s at a rate of 30 frames/s. As respiration can affect venous return and influence blood flow, we advised the subjects to breathe gently and not to perform any Valsalva-like maneuver or move the body or legs during the measurements.

VPT (expressed in m/s^2^) were measured by the same researcher at three locations: plantar aspect of the hallux, the base of the little toe, and the heel of the right leg following the protocol mentioned in the international standard ISO 13091-1 [37]. A commercially available vibrotactile perception meter, VPM (HVLab, University of Southampton, UK) was used for this purpose. The test locations were randomized for each participant. During measurements, the subjects sat comfortably upright with their eyes open, right hand on the right thigh and the left hand holding the switch of the VPT device. Their left foot rested on the wooden floor and the right foot rested on a level with the vibrating probe that was fixed at a height of about 4 cm above the floor. Upon instruction, the subjects placed the measurement site of right foot over a cylindrical vibrating probe of 6 mm in diameter with a surround (probe-surround gap 2 mm) contact force of approximately 2 N. The applied force was displayed on the digital scale of the vibrotactile perception meter. The vibratory stimulus was delivered at the test frequency of 31.5 Hz perpendicular to the skin surface, via the stimulating probe [37]. According to the method referred to as a von Bekesy up/down psychophysical algorithm, at a constant rate of 3 dB/s, the vibration magnitude increased until the stimulus was perceived (when the subject was instructed to press the response button by the left thumb) and then decreased until the stimulus could no longer be felt (when the subject was asked to release the response button) [38]. This sequence was repeated at least six times. The VPT value was calculated as the mean of the peak (ascending) and trough (descending) thresholds. During measurements, the vibrometer display was kept out of the subject’s sight.

Room temperature and foot skin temperature were measured by using digital thermistors (SZL-64, Technol seven, Kanagawa, Japan) with a measurement accuracy of ±0.15 °C, connected to a scanner (X115, Technol seven, Kanagawa, Japan) and high-accuracy data logger (K730, Technol seven, Kanagawa,, Japan).

### 2.5. Data Processing and Statistical Analyses:

For scanning of SBF, a circular region on the dorsum immediately proximal to the metatarsophalangeal joints and between the edges of the right foot was chosen (260–310 pixels, depending on the size of the foot). The captured images were processed and analyzed by the same assessor using the LSFG analysis software version 3.0.36.0 (Softcare Co., LTD., Fukuoka, Japan).

The values of SBF measured twice before intervention were averaged for each of the 4 exposure conditions and considered as the baseline values for the corresponding conditions. Also, for the balance and mobility tests, the results were calculated as the average of two trials.

The continuous variables of this study showed non-normal distributions as verified by Kolmogorov-Smirnov and Shapiro-Wilk tests. Statistical analyses were performed after logarithmic transformation of the collected data and the results have been reported in the original scale after back transformation. The continuous variables were expressed as geometric mean and corresponding 95% CI in the text, and tables and figures. Repeated measures analysis of variance (ANOVA) was used to test for the effects of measurement time points and exposure conditions. SBF and VPT data showed a significant time × condition interaction (*p* < 0.001). Further analyses with repeated measures ANOVA was conducted at each time point between conditions for the measured variables with Bonferroni adjustments in the level of significance for multiple comparisons. All statistical tests were considered as two-tailed, and the significance level was set at *p* < 0.05. The software package SPSS version 22 for Windows (SPSS Inc., Chicago, IL, USA) was used to perform the statistical analyses.

### 2.6. Ethical Statement

All subjects provided written informed consent before they participated in this study. The study was conducted in accordance with the Declaration of Helsinki, and the protocol was approved by the relevant institutional review board of Yamaguchi University School of Medicine (approval no. H30-057-2, dated 25-09-2019).

## 3. Results

**Figure 2:**
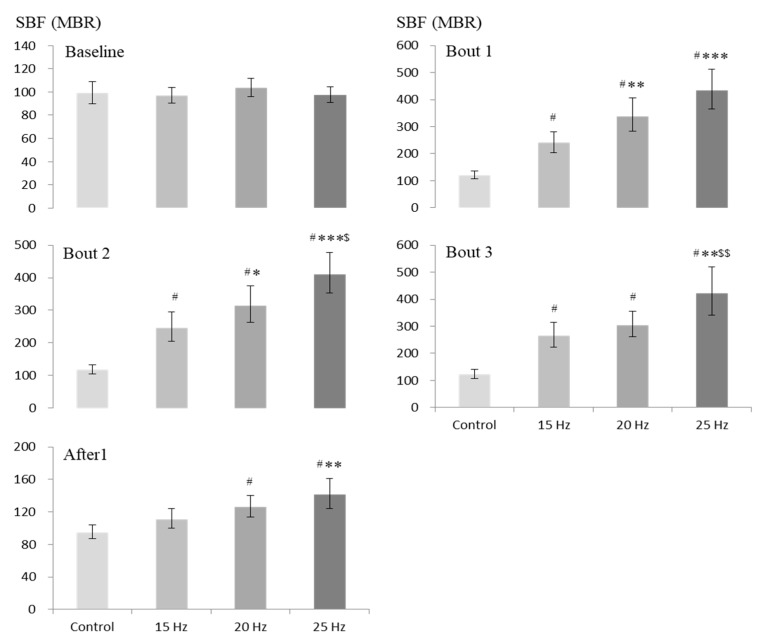
Comparison of SBF (MBR) between 4 exposure conditions at baseline (before), during (bouts 1, 2 and 3), and after exposure (n = 30). Values are shown as geometric mean and 95% CI (shown as error bars). Levels of significant differences from the corresponding values by repeated measures ANOVA with adjustments for multiple comparisons by Bonferroni corrections: ^#^ *p* < 0.001 versus control; ^***^ *p* < 0.001, ^**^ *p* < 0.005 and ^*^ *p* < 0.05 versus 15 Hz; ^$$^ *p* < 0.01 and ^$^ *p* < 0.05 versus 20 Hz.

Figure 2 displays the values of SBF obtained before, during (3 time points) and after exposure under each of the four exposure conditions. Data analysis including only the baseline values of SBF before intervention revealed that the corresponding values under the exposure conditions of 15, 20 and 25 Hz did not differ significantly from the control value. 

During intervention, compared to the corresponding control condition, WBV was associated with a significant increase in SBF under all 3 vibration exposure conditions for all 3 bouts (in comparison with the corresponding control values, about 126%–150%, 171%–206% and 300%–322% higher mean SBF under 15, 20 and 25 Hz conditions, respectively; *p* < 0.001) (Figure 2). When the comparison was limited between vibration exposure conditions only, compared to the corresponding values under the 15 Hz condition, the increase in SBF showed significantly higher values for first 2 bouts under the 20 Hz condition (*p* < 0.05 to 0.005), and for all 3 bouts under the 25 Hz condition (*p* < 0.005 to 0.001). When the comparison was made between the latter conditions during exposure, a significantly greater increase in SBF was observed under the 25 Hz exposure condition for second and third bouts during intervention (*p* < 0.05 to 0.01).

After the cessation of intervention, compared to the control condition, the significant increase in SBF disappeared under the 15 Hz condition, but remained significantly elevated under two other vibration exposure conditions (*p* < 0.001). However, the extent of the latter increase in SBF was much lesser (26% and 49% higher mean SBF under 20 and 25 Hz conditions, respectively) than that observed during intervention (Figure 2). When the comparison was made with the 15 Hz condition, SBF showed a significantly higher value under the 25 Hz exposure condition only (*p* < 0.005). But after intervention, no statistically significant difference in SBF was observed between 20 Hz and 25 Hz exposure conditions.

**Figure 3:**
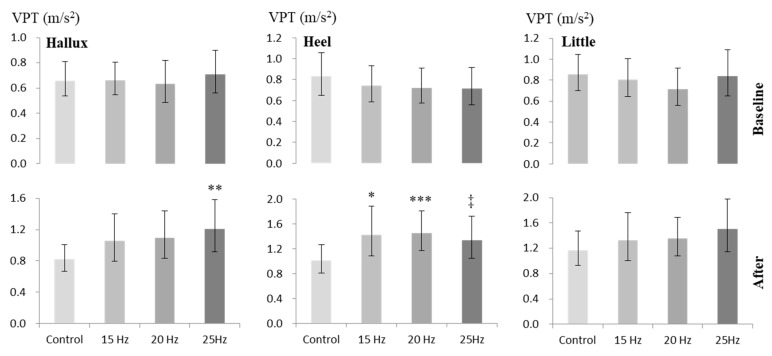
VPT (dB) at the hallux (left panel), heel (middle panel) and little (right panel) finger at baseline (upper panel) and after intervention (lower panel) under 4 different exposure conditions (n = 29). Values are presented as geometric mean and 95% CI (shown as error bars). Levels of differences from the corresponding control values by repeated measures ANOVA with adjustments for multiple comparisons by Bonferroni corrections: ^***^ *p* < 0.005, ^**^ *p* < 0.01, ^*^ *p* < 0.05; ^‡^ *p* = 0.06.

The values of VPT obtained before and after intervention under 4 exposure conditions at three test locations of right plantar foot have been presented in Figure 3. Comparison between the before-intervention values at three test locations did not reveal any significant difference between the exposure conditions.

After intervention, compared to the control value at the hallux, VPT increased significantly under 25 Hz vibration exposure condition only (*p* < 0.01). Compared to the control value at the heel, the increase in VPT was significant under 15 Hz (*p* < 0.05) and 20 Hz (*p* < 0.005) exposure conditions, and the increase was notable under the 25 Hz condition (although did not reach the level of significance; *p* = 0.063). At the little finger, the after-intervention values of VPT under vibration conditions did not differ significantly from the control value. When multiple comparisons were made between the after-intervention values of VPT obtained under 15, 20 and 25 Hz vibration exposure conditions, no significant differences could be revealed between those values.

Table 1 represents the effects of the intervention under 4 exposure conditions on the balance and mobility tests that were assessed by OLS test (eyes open and closed), PW test, TUG test, and CS-30 test. As the results show, the before-exposure values for any of the tests did not differ significantly between the four exposure conditions. Also, after intervention, no statistically significant WBV-induced improvements in those tests could be revealed from comparison of the values of different tests with the corresponding control values.

## 4. Discussion

In this study, we characterized the specific patterns of acute responses in peripheral blood flow, plantar cutaneous sensitivity, and balance in older adults induced by short-term exposure to WBV of 15, 20 and 25 Hz, the magnitudes of which were selected considering the exposure limit recommended by the existing international standards [23,24]. As observed, the applied vibration induced an improvement in peripheral SBF, but caused a decrease in plantar cutaneous sensation. However, we could not observe any positive change in body balance among the older adult subjects.

In the current study, we used a knee flexion angle of 30° during intervention, in order to minimize the transmission of side-alternating vibration to the head. Furthermore, we used an intermittent intervention protocol with 3 min of cumulative WBV exposure considering the possible muscle fatigue and suppression of tonic vibration reflex from longer duration of exposure [39,40]. We also considered the relevant exposure limits recommended by ISO 2631-1 and EU Directive 2002/44/EU [23,24].

As reported in the literature, the natural resonance frequencies of the human head, chest and abdomen range between 2 and 11 Hz [41]. Prolonged exposure of human body to vibration, especially at its resonant frequency may cause undue stress and discomfort [42]. On the other hand, occupational exposure to WBV with the dominant frequency range of 28 Hz and 40 Hz has been reported to cause impairments in peripheral circulation and associated symptoms among the exposed workers [43]. As recommended by the international standard ISO 2631-1, human exposure to WBV should not include high levels of vibration [23]. The standard also suggests a health guidance caution zone above which there exist potential risks to health. Furthermore, the EU Directive 2002/44/EU states that people with regular occupational exposure to WBV should not be exposed to the vibration level exceeding an A(8) value of 1.15 m/s^2^ rms [24]. On the other hand, for exposures between 1 min and 10 min—the duration commonly being used for exposure to devices producing WBV—the upper boundary of the caution zone is assumed to be at the frequency-weighted acceleration of 6.0 m/s^2^ rms and the lower boundary, at 3.0 m/s^2^ rms [44]. Considering all this, we decided to use the vibration frequencies of 15, 20 and 25 Hz with the same displacement, which resulted in the corresponding A(8) values (expressed in m/s^2^ rms) of 0.76, 1.12, and 1.41, respectively. In this study, using the same feet position on the vibration platform and the same amplitude of vibration for all three frequencies led to the generation of a level of vibration magnitude that exceeded the recommended A(8) value at 25 Hz. However, we believe that subjects’ posture on the platform with flexed knees caused partial reduction of the mechanical energy of WBV transmitted to the body through the feet [39].

Exposure to different frequencies of WBV causes stimulations of specific skin mechanorecerptors that are reflected by the changes in VPT representing the relevant mechanoreceptors. The exposure frequencies used in our study should have stimulated the Meissner corpuscles which are the most common mechanoreceptors found in the glabrous skin [45]. These mechanoreceptors are selectively sensitive to low-frequency vibrations between 5 and 40 Hz, with a peak sensitivity around 30 to 40 Hz [46]. For investigation of vibrotactile perception mediated by the Meissner corpuscles, a vibration exposure frequency like 31.5 Hz has been suggested [23]. Therefore, in this study, we investigated the VPT at the test frequency of 31.5 Hz. 

As revealed, WBV generated at 15, 20 and 25 Hz caused a significant increase in foot SBF both during and/or after the exposure comparing with the corresponding control condition. Such enhancements in SBF are caused by vibration stimulation-induced interactions of neural signals, hormones and mediators with the involvement of both local and central vasoregulatory mechanisms [13,16,39,47,48]. It has been postulated that exposure to WBV generates vibration tonic reflexes which increase muscle metabolic demand and oxygen consumption with rhythmic contraction and relaxation of the precapillary sphincters and subsequent vasodilation [13,15]. Such exposure also results in a pulsatile blood flow and shear stress of endothelial cells which can cause enhanced production of nitric oxide, a potent vasodilator [16]. The role of other potent vasodilatory agents like prostaglandins has also been suggested in the literature [47]. These factors probably played roles in the observed vibration-induced vasodilation and subsequent increases in peripheral SBF.

Our findings of WBV-induced increase in foot SBF are comparable with those of Herrero et al. (WBV frequency, 10, 20 and 30 Hz), Johnson et al. (26 Hz), and Lythgo et al. (5 Hz to 30 Hz), who exposed study populations to side-alternating vibration platform and observed an increase in lower extremity blood flow, especially at vibration exposure frequencies ranging between 20 to 30 Hz [15,16,47]. However, in all these studies, the peak vibration magnitude under most of the exposure conditions largely exceeded the recommended value, and the applications of such high levels of WBV into practice are questionable [13]. On the other hand, our findings contradict the findings of two studies by Lohman et al., who did not observe such improvements in peripheral circulation from exposure to active WBV [19,49]. However, this might have been caused by the fact that those studies by Lohman et al. used a comparatively high frequency (50 Hz) with high magnitudes of WBV (A(8) values 6.19–7.43 m/s^2^ rms) [13]. In contrast, the same group of researchers found a significant increase in SBF while using a relatively lower vibration frequency (30 Hz) with lower magnitudes of WBV (A(8) values 2.01–2.41 m/s^2^ rms) [48]. As observed in our study, the increase in SBF was significantly greater under the condition of 25 Hz than that at 20 or 15 Hz, but the responses did not appear to differ largely between 20 and 25 Hz exposure conditions after intervention. However, the fact that the peak vibration magnitude exceeded the recommended safe exposure limit at 25 Hz and SBF remained significantly elevated after cessation of vibration exposure at 20 Hz, these indicate the efficacy and practical applicability of exposure to WBV at 20 Hz for increasing SBF of the lower extremity in older adults.

In this study, the observed increases in VPT did not seem to have a direct influence on the balance and mobility of the older study subjects as exposure to different interventions did not produce any significant change in the relevant test results. As we did not observe any vibration-induced improvements in balance and functional mobility, one possible explanation might be the level of vibration magnitude used in our study, which was probably not high enough, as in a number of other studies [17,18], to induce such effects. Another issue may be the fact that the older subjects enrolled in this study were relatively healthy and physically active with good baseline balance and mobility, and there was probably little scope for further improvements in the latter. It should be mentioned here that the available reports investigating the effects of WBV on the cutaneous mechanoreceptor perception of feet and balance generated considerable debate with conflicting results. Sonza et al. reported that for young healthy people, a single 10-min exposure to WBV of 42 Hz decreased the touch-pressure and vibration sensitivity of the plantar foot, and up to 2.5 h was required for the sensory system to return to baseline values [50]. In an investigation, Pollock et al. exposed young healthy individuals to WBV at 30 Hz, and in line with our findings, observed a reduction in foot cutaneous sensation with no positive effects on balance from the exposure [29]. In contrast, Schlee et al. found that exposure of young subjects to WBV at 27 Hz caused improvements in balance and movement control despite a decrease in plantar foot sensitivity [27]. Considering the findings observed by us and those of the others, it seems difficult to explain how plantar cutaneous sensation contributes specifically to the observed responses in balance and functional mobility. Rittweger suggested that improvements in balance among older people may be due to the training effects during WBV, rather than stimulation of sensory structures as their sensitivity decreases with age [51]. Therefore, it seems reasonable to postulate that neuromuscular mechanisms (for example, increase in muscle spindle activation) probably have predominant roles versus an increase in the foot sensitivity in vibration-induced improvements in body balance.

In contrast to short-term exposure to WBV, a number of studies demonstrated significant positive effects of repeated long-term exposure to WBV on balance and functional mobility among older adults. For example, del Pozo-Cruz et al. demonstrated a significant improvement in both foot VPT and postural stability from long-term exposure to 6–8 min of intermittent and/or continuous WBV (20 Hz; 2 times/week for 12 weeks) [28]. Runge et al. exposed community-dwelling ambulatory seniors to side-alternating WBV (27 Hz; 3 times/week for 8 weeks) and observed 18% improvements in chair rising times in the WBV group compared to the control group [52]. Bruyere et al. observed a significant decrease in TUG test time among elderly nursing home residents from exposure to side-alternating WBV (combination of 10 Hz and 26 Hz; 3 times/week for 6 weeks) [53]. In a study, Gusi et al. reported a 29% improvement in SLS test (with eyes closed) from exposure to side-alternating WBV (12.6 Hz; 3 times/week for 8 months) among a group of physically untrained post-menopausal women [54]. In contrast, Beck and Norling in a randomized controlled trial exposed a group of postmenopausal women to side-alternating WBV (12.5 Hz; twice/week for 8 months) and could not observe vibration-induced improvements in balance among the study participants [55]. Therefore, the effects of WBV-intervention on balance remain inconclusive and the effective vibration parameters for the purpose remain uncertain. However, as there is substantial evidence for improvements in balance among older adults from repeated long-term exposure to WBV, future studies should investigate the changes in balance and mobility from repeated long-term exposure to WBV at different frequencies with magnitudes considering the recommended limits for such exposure.

There are some potential limitations to the findings of the current study. This study was conducted among apparently healthy older adults and the current findings may not be generalizable to older patient populations. The magnitudes of WBV were chosen in line with the recommendations by ISO 2631-1 and EU Directive 2002/44/EU which have been developed to assess the health effects of chronic long-term occupational exposure of human subjects to WBV [23,24]. Therefore, the question may arise whether these standards are applicable to the current study population. It has been suggested that the safety limits given in ISO 2631–1 may serve a guideline in settling vibration frequencies and amplitudes, and the duration of a single training session with exposure to WBV [56]. Furthermore, exposure to WBV in sports and exercise centers and for treatment or rehabilitation purpose would need long-term chronic interventions, which justifies the applicability of those standards for such exposure [13]. In future, these standards should clearly state the relevance and applicability of the existing international standards for therapeutic exposure to WBV. Another limitation is that due to the current study design, we could not follow up SBF for more than 1 min after cessation of intervention as we measured the after-effects of WBV on VPT which was also followed by the balance tests. It should also be noted that we did not attempt to investigate the sex differences in the observed findings of our study. However, as sex is known to influence different human physiological responses [57], future studies should examine how the responses to the varying WBV frequencies with different magnitudes differ across the sexes. Also, we did not record the history of falls among the study participants who were relatively healthy. However, we believe that this does not influence the outcome of our study as inclusion of such subjects and any probable improvements in balance among them, as observed by others [58], would mean an underestimation of the current results. However, as peripheral circulation is important for maintenance of muscle health and reducing orthostatic intolerance, especially in older persons, future studies should assess peripheral blood flow as well as use questionnaire addressing the risk of falls when using WBV interventions. Lastly, we could not reveal any acute changes in balance and mobility among older subjects from short-term exposure to WBV; the changes from long-term exposure to repeated WBV may be different from the observed effects in this study. However, this needs to be investigated in future among older subjects with impaired balance and mobility, considering the recommended limits for such exposure.

## 5. Conclusions

Acute exposure of older adults to WBV of 15, 20 and 25 Hz with the same peak-to-peak displacement of 4 mm induced an improvement in peripheral SBF and caused a decrease in plantar cutaneous sensation without producing any positive changes in body balance. For the purpose of increasing peripheral SBF, exposure to WBV of 20 Hz with a peak-to-peak displacement of 4 mm might be considered as it generates a vibration magnitude that is within the recommended limit by ISO 2639-1 and EU Directive 2002/44/EU. Furthermore, we speculate that vibration magnitudes within the recommended limit able to enhance peripheral SBF might not be sufficient in inducing improvements in balance among older adults. However, the current findings might be useful in establishing the strategies for proper application of WBV and developing new guidelines by the relevant standards on safe and effective use of vibration-related parameters, for therapeutic exposure of human subjects to WBV in various pathological conditions.

## Figures and Tables

**Table 1:** The results of balance and mobility tests obtained before and after intervention under different exposure conditions. Values are shown as geometric mean (95% CI) (n = 30).

Test	Before/after	Control	15 Hz	20 Hz	25 Hz
OLS, eyes	Before	25.1 (18.7–33.6)	28.8 (21.5–38.6)	27 (20.3–36.0)	26.1 (19.1–35.6)
open (s)	After	29.6 (23.5–37.4)	26.8 (19.2–37.4)	29 (22.9-36.6)	26.8 (19.4-37.1)
OLS, eyes	Before	3.7 (2.9–4.8)	3.3 (2.6–4.2)	4.0 (3.2–4.9)	3.4 (2.7–4.1)
closed (s)	After	3.7 (2.8–4.7)	3.6 (2.8–4.7)	4.3 (3.4–5.5)	3.8 (3.1–4.6)
PW	Before	4.6 (4.3–4.9)	4.5 (4.3–4.8)	4.7 (4.3–5.0)	4.5 (4.3–4.8)
(s)	After	4.6 (4.3–4.9)	4.5 (4.3–4.8)	4.4 (4.2–4.7)	4.4 (4.2–4.7)
TUG	Before	6.4 (6.1–6.7)	6.4 (6.0–6.7)	6.3 (6.0–6.7)	6.2 (5.9–6.6)
(s)	After	6.4 (6.1–6.8)	6.4 (6.0–6.7)	6.3 (6.0–6.6)	6.2 (5.9–6.6)
CS-30	Before	18.1 (16.4–19.9)	18.7 (17.1–20.5)	18.9 (17.0–21.1)	19.1 (17.4–21.0)
(repetitions)	After	18.6 (16.9–20.5)	19.1 (17.2–21.0)	19.1 (17.2–21.3)	20.0 (18.2–22.0)
